# Flora of Wounds in Relation to Secondary Suture

**Published:** 1918-11

**Authors:** 


**Flora of Wounds in Relation to Secondary Suture.** Fleming and Janes, Brit. Med. Jour., Feb. 9, found in 55 unsuccessful cases, streptococci, spore-bearing bacilli and anaerobes digesting the tissues and yielding gas having the odor of rotten eggs. On the other hand, when the culture did not yield these microorganisms, healing occurred even with 20-30 bacteria to a field. The authors, therefore, urge cultural studies before undertaking secondary suture. (Note: This communication is in line with various others based on war experience, dealing with the most diverse problems but all having the common point that we must get away from the conception of non-specific bacteria. The determination of the exact microorganism is of great practical importance.

Some of these studies even indicate that the differential diagnosis may be made clinically.

